# Supplementary material for: Analysis of the complete genome sequence of a marine-derived strain Streptomyces sp. S063 CGMCC 14582 reveals its biosynthetic potential to produce novel anti-complement agents and peptides
Source: PeerJ. 2019 Jan 3;7:e6122. doi: 10.7717/peerj.6122 (PMC6321760; doi:10.7717/peerj.6122)
Supplement: Supplemental Information 1 — This files include the supplementary figures and tables. [file peerj-07-6122-s001.docx]

Supplementary materials for

**Analysis of the complete genome sequence of** **a marine streptomycete *Streptomyces* sp. S063 CGMCC 14582 reveals its potential to produce novel secondary metabolites including anti-complement agents**

Liang-Yu Chen^1^, Hao-Tian Cui^2^, Chun Su^3*^, Feng-Wu Bai^2^ and Xin-Qing Zhao^2*^

^1^School of Life Science and Biotechnology, Dalian University of Technology, Dalian 116023, China.

^2^State Key Laboratory of Microbial Metabolism and School of Life Science and Biotechnology, Shanghai Jiao Tong University, Shanghai 200240, China.

^3^National Engineering Laboratory for Resource Developing of Endangered Chinese Crude Drugs in Northwest China, College of Life Sciences, Shaanxi Normal University, Xi’an 710119, China.

*Corresponding authors:

Dr. Chun Su. E-mail: suchun@snnu.edu.cn. Tel: +86-29-85310581. Fax: +86-29-85310623; Prof. Dr. Xinqing Zhao, E-mail: xqzhao@sjtu.edu.cn. Tel: +86-21-34206673. Fax: +86-21-34208028.

# Supplementary tables

#### Table S1 Culture medium used in this study

| Name | Composition (g/L) | pH |
| --- | --- | --- |
| A1 | yeast extract 4, soluble starch 10, peptone 2, sea salt 28 | 7.0 |
| TSB | tryptone 17, soy peptone 3, glucose 2.5, K_2_HPO_4_ 2.5 | 7.2 |
| MS | starch or cellulose 10, K_2_HPO_4_ 0.5, MgSO_4_ 0.5, NaCl 0.5，KNO_3_ 1, agar 20 | 7.0 |
| M2 | soluble starch 20, glucose 10, peptone 5, yeast extract 5, CaCO_3_ 5 | 7.0 |
| M3 | soluble starch 20, soybean powder 25, NH_4_Cl 2, NaCl 2, K_2_HPO_4_ 0.5, CaCO_3_ 5 | 7.2 |
| M8 | malt extract 40, yeast extract 4, glucose 2, NH_4_Cl 0.8, Na_2_HPO_4_ 1 | 6.0 |
| M9 | sucrose 20, soybean powder 10, corn syrup 10, KCl 8 | 6.5 |
| M12 | glucose 100, yeast extract 10, CaCO_3_ 2 | 6.8 |
| M17 | glucose 10, soy flour 10, corn syrup 10, glycerol 5, yeast extract 5, NaCl 5, CaCO_3_ 2 | 5.7 |
| M19 | soluble starch 20, cotton seed powder 5, yeast extract 2.5, NaCl 1, K_2_HPO_4_ 0.75, MgSO_4_·7H_2_O 1, CaCO_3_ 3 | 7.5 |
| M22 | malt extract 35, corn starch 30, corn syrup 15, cotton seed powder 15, CaCO_3_ 2 | 7.5 |

#### Table S2 Primers used in this study

| Name | Sequence |
| --- | --- |
| apra F | TGTAGGCTGGAGCTGCTTC |
| apra R | TATTCCGGGGATCCGTCGAC |
| 4-k1 F | GCCTGCAGGTCGACTCTAGA CGACGGAAACACGGGAGAATCA |
| 4-k1 R | CGAAGCAGCTCCAGCCTACA TGAAGTGCTCCACGAACAGTCC |
| 4-k3 F | TCGACGGATCCCCGGAATAT TACGGAGTCGTGACGTGTGC |
| 4-k3 R | AATTCGAGCTCGGTACCCGG CGGAGCCGTGAACTGACACT |
| 23-k1 F | GCCTGCAGGTCGACTCTAGA CGCAAGTACCACAAGCACCTGA |
| 23-k1 R | CGAAGCAGCTCCAGCCTACA TGTCGGAGTTCCTGGGCTGA |
| 23-k3 F | TCGACGGATCCCCGGAATAT AGATGCTGCTGCGCACTCT |
| 23-k3 R | AATTCGAGCTCGGTACCCGG CTACCAGACCACCCGGAGC |
| 25-k1 F | GCCTGCAGGTCGACTCTAGA GCACGGATCGCCGAATGGAT |
| 25-k1 R | CGAAGCAGCTCCAGCCTACA TGGTGGGCCAGCATCTCGAT |
| 25-k3 F | TCGACGGATCCCCGGAATAT GCGGTGATCTCATCGATTCGG |
| 25-k3 R | AATTCGAGCTCGGTACCCGG GGGGCTCATGAACCGGCG |
| 28-k1 F | GCCTGCAGGTCGACTCTAGA ATGAGCACCCAGACAGCCCA |
| 28-k1 R | CGAAGCAGCTCCAGCCTACA TTCAGGCCTGCTGGTGCAG |
| 28-k3 F | TCGACGGATCCCCGGAATAT GGTCAGCCGTTGAACGTGTCC |
| 28-k3 R | AATTCGAGCTCGGTACCCGG ATGCGCATGCGACCCACC |
| 4-773 F | ACGACGTCCTCGCCACCGGACTGTTCGTGGAGCACTTCA TGTAGGCTGGAGCTGCTTC |
| 4-773 R | GACGGCCGGGGTGCGGCGCGCACACGTCACGACTCCGTA TATTCCGGGGATCCGTCGAC |
| 4-p18 F | ACCGAACGACCGGGTGGCCAGTGTCAGTTCACGGCTCCG CCGGGTACCGAGCTCGAATT |
| 4-p18 R | CTCTCTCGGTTGTCGAATGATTCTCCCGTGTTTCCGTCG TCTAGAGTCGACCTGCAGGC |
| 23-773 F | AGCCCCTTCGGGTCGCGGGTCAGCCCAGGAACTCCGACA TGTAGGCTGGAGCTGCTTC |
| 23-773 R | AGGGGTCGACGGAGACGTACAGAGTGCGCAGCAGCATCT TATTCCGGGGATCCGTCGAC |
| 23-p18 F | ACCTCCCGCAGCCCGCGGCGGCTCCGGGTGGTCTGGTAG CCGGGTACCGAGCTCGAATT |
| 23-p18 R | GGACATCTCCGACAGCTTCAGGTGCTTGTGGTACTTGCG TCTAGAGTCGACCTGCAGGC |
| 25-773 F | AGAGGCGCTGGAAGGCCTGATCGAGATGCTGGCCCACCA TGTAGGCTGGAGCTGCTTC |
| 25-773 R | GTGGGGACGGCAGGCCGTCCGAATCGATGAGATCACCGC TATTCCGGGGATCCGTCGAC |
| 25-p18 F | CATCACCCCCGCGGCCCCGCCCGCCGGTTCATGAGCCCC CCGGGTACCGAGCTCGAATT |
| 25-p18 R | CGCCGGTGACGTCGCCGCGATCCATTCGGCGATCCGTGC TCTAGAGTCGACCTGCAGGC |
| 28-773 F | ATGCGGTGGACCCCCGCACCCTGCACCAGCAGGCCTGAA TGTAGGCTGGAGCTGCTTC |
| 28-773 R | GCGCACCGGGCCCGACCCGGACACGTTCAACGGCTGACC TATTCCGGGGATCCGTCGAC |
| 28-p18 F | CTCGGCGGAGGTCCAGCTCAGGGTGGGTCGCATGCGCAT CCGGGTACCGAGCTCGAATT |
| 28-p18 R | GGCGGTCGGTGCCTCGCGGTGGGCTGTCTGGGTGCTCAT TCTAGAGTCGACCTGCAGGC |
| 4yan F | ACACGATCATCGACCACACCTG |
| 23yan F | CATCGAGGTCGAGCACCTGTT |
| 25yan F | TACGGCAAGCCGTACTGGA |
| 28yan F | CGTGATCGAACTGCTGCGC |
| 773yan R | ATCCATTGCCCCTGCCACCT |

Note: The underlined letters indicate the homologous regions.

#### Table S3 General features of *Streptomyces* sp. S063

| Items | Description |
| --- | --- |
| Classification | Domain *Bacteria* |
|  | Phylum *Actinobacteria* |
|  | Class *Actinobacteria* |
|  | Order *Actinomycetales* |
|  | Family *Streptomycetaceae* |
|  | Genus *Streptomyces* |
|  | Species *Streptomyces badius* |
|  | Strain: S063 |
| Gram stain | Positive |
| Motility | Motionless |
| Pigmentation | Yellowish white |
| Cell shape | Branched mycelia |
| Temperature range (°C) | 4-37, optimally at 28 |
| Biotic relationship | Free living |
| Salinity (w/v) | 0.5-9.5%, optimum 3.5% NaCl* |
| Relation to oxygen | Aerobic |

Note: *, TSB medium contains 0.5% (w/v) NaCl.

#### Table S4 Number of genes associated with general COG functional categories

| Code | No. | Percentage | Description |
| --- | --- | --- | --- |
| J | 208 | 2.93 | Translation, ribosomal structure and biogenesis |
| A | 4 | 0.06 | RNA processing and modification |
| K | 683 | 9.61 | Transcription |
| L | 233 | 3.28 | Replication, recombination and repair |
| B | 2 | 0.03 | Chromatin structure and dynamics |
| D | 53 | 0.75 | Cell cycle control, Cell division, chromosome partitioning |
| V | 129 | 1.81 | Defense mechanisms |
| T | 429 | 6.03 | Signal transduction mechanisms |
| M | 269 | 3.78 | Cell wall/membrane biogenesis |
| N | 8 | 0.11 | Cell motility |
| U | 46 | 0.65 | Intracellular trafficking and secretion |
| O | 159 | 2.24 | Posttranslational modification, protein turnover, chaperones |
| C | 294 | 4.14 | Energy production and conversion |
| G | 456 | 6.50 | Carbohydrate transport and metabolism |
| E | 462 | 6.54 | Amino acid transport and metabolism |
| F | 112 | 1.58 | Nucleotide transport and metabolism |
| H | 231 | 3.25 | Coenzyme transport and metabolism |
| I | 264 | 3.71 | Lipid transport and metabolism |
| P | 251 | 3.53 | Inorganic ion transport and metabolism |
| Q | 243 | 3.42 | Secondary metabolites biosynthesis, transport and catabolism |
| R | 753 | 10.59 | General function prediction only |
| S | 423 | 5.95 | Function unknown |
| - | 2,740 | 38.54 | Not in COGs |

Note: The percentage is calculated based on the total number of protein coding genes in the genome.

#### Table S5 Genome comparison of *Streptomyces* sp. S063 and the subspecies of *S. griseus* using OrthoANIu values

| Organism/Name | Strain | Assembly | Size (Mb) | Scaffolds | Gene | OrthoANIu value (%) | AAL (bp) # |
| --- | --- | --- | --- | --- | --- | --- | --- |
| *S. griseus subsp. griseus* | NBRC 13350 | GCA_000010605.1 | 8.55 | 1* | 7,284 | 89.9 | 4,055,388 |
| *S. griseus* | BIG105 | GCA_001715295.1 | 6.87 | 16** | 5,996 | 82.45 | 2,763,591 |
| *S. griseus* | NRRL B-2165 | GCA_001723115.1 | 8.10 | 1,108** | 7,277 | 90.02 | 3,607,713 |
| *S. griseus subsp. griseus* | NRRL B-2682 | GCA_001723125.1 | 7.96 | 1,244** | 7,202 | 90.22 | 3,539,928 |
| *S. griseus* | S4-7 | GCA_000932225.2 | 7.69 | 3 | 6,927 | 93.53 | 4,320,872 |
| *S. griseus* | DSM 40236 | GCA_900105705.1 | 8.63 | 4 | 7,381 | 89.87 | 4,138,770 |
| *S. griseus subsp. rhodochrous* | NRRL B-2931 | GCA_000718205.1 | 8.03 | 67 | 7,120 | 88.7 | 3,818,413 |
| *S. griseus subsp. rhodochrous* | NRRL B-2930 | GCA_000721205.1 | 8.03 | 85 | 7,114 | 88.79 | 3,794,648 |
| *S. griseus subsp. griseus* | NRRL F-2227 | GCA_000721575.1 | 8.04 | 86 | 7,055 | 88.75 | 3,829,922 |
| *S. griseus subsp. griseus* | NRRL F-5144 | GCA_000719355.1 | 7.85 | 89 | 7,110 | 93.6 | 4,267,182 |
| *S. griseus subsp. rhodochrous* | NRRL B-2929 | GCA_001270675.1 | 8.03 | 102 | 7,125 | 88.79 | 3,802,556 |
| *S. griseus subsp. griseus* | NRRL B-2307 | GCA_000717375.1 | 7.20 | 135 | 6,391 | 78.82 | 2,288,652 |
| *S. griseus subsp. rhodochrous* | NRRL B-2932 | GCA_000718235.1 | 7.84 | 142 | 6,808 | 88.86 | 3,844,738 |
| *S. griseus subsp. griseus* | NRRL F-5618 | GCA_000719435.1 | 7.10 | 165 | 6,247 | 78.82 | 2,349,132 |
| *S. griseus subsp. griseus* | NRRL WC-3480 | GCA_000717795.1 | 8.33 | 166 | 7,177 | 89.9 | 4,021,963 |
| *S. griseus subsp. griseus* | NRRL B-2621 | GCA_000721035.1 | 7.43 | 174 | 6,717 | 79.43 | 2,402,716 |
| *S. griseus subsp. rhodochrous* | NRRL B-1691 | GCA_000719035.1 | 8.09 | 228 | 7,124 | 88.75 | 3,798,650 |
| *S. griseus subsp. griseus* | NRRL B-2621 | GCA_000718525.1 | 7.43 | 239 | 6,731 | 79.49 | 2,414,674 |
| *S. griseus subsp. griseus* | NRRL F-5621 | GCA_000719495.1 | 7.34 | 240 | 6,438 | 78.75 | 2,257,522 |
| *S. griseus subsp. griseus* | NRRL WC-3645 | GCA_000720255.1 | 9.89 | 265 | 9,047 | 78.14 | 2,290,396 |
| *S. griseus subsp. griseus* | NRRL WC-3066 | GCA_001509565.1 | 7.15 | 287 | 6,322 | 78.82 | 2,288,023 |
| *S. griseus subsp. rhodochrous* | NRRL B-2933 | GCA_000717555.2 | 7.84 | 4,281 | - | 89.08 | 2,825,857 |

Note: #AAL, Average aligned length; *, complete level; **, scaffold level; the rest genomes are contig level. OrthoANIu value and AAL value are calculated by comparing *Streptomyces* sp. S063 with the selected strains.

#### Table S6 Deduced gene functions of *Cluster17*

| Gene | Identity | Coverage | Subject gene | Function |
| --- | --- | --- | --- | --- |
| *gene4741* | 85 | 61.8 | BAG19226.1 | AmfT protein |
| *gene4742* | 100 | 100.0 | BAG19225.1 | AmfS protein |
| *gene4743* | 83 | 95.8 | BAG19224.1 | membrane translocator |
| *gene4744* | 86 | 97.1 | BAG19223.1 | membrane translocator |
| *gene4745* | 92 | 100.0 | BAG19222.1 | transcriptional regulator |

#### Table S7 Deduced gene functions of *Cluster8*

| Gene | Identity | Coverage | Subject gene | Function |
| --- | --- | --- | --- | --- |
| *gene975* | 98 | 100.0 | EFE73293.1 | transmembrane-transport protein |
| *gene976* | 98 | 98.4 | EFE73294.1 | TetR-family transcriptional regulator |
| *gene977* | 94 | 100.0 | EFE73295.1 | conserved hypothetical protein |
| *gene978* | 98 | 100.0 | EFE73296.1 | oxidoreductase |
| *gene979* | 99 | 100.0 | EFE73297.1 | argininosuccinate lyase ArgH |
| *gene980* | 99 | 100.0 | EFE73298.1 | argininosuccinate synthase ArgG |
| *gene981* | 97 | 100.0 | EFE73300.1 | secreted protein |
| *gene982* | 100 | 100.0 | EFE73301.1 | arginine repressor |
| *gene983* | 96 | 99.0 | EFE73302.1 | N2-acetyl-L-ornithine: 2-oxoglutarate aminotransferase ArgD |
| *gene984* | 97 | 100.0 | EFE73303.1 | N-acetylglutamate kinase ArgB |
| *gene985* | 96 | 100.0 | EFE73304.1 | N2-acetyl-L-ornithine: L-glutamate N-acetyltransferase ArgJ |
| *gene986* | 97 | 99.7 | EFE73305.1 | N-acetyl-gamma-glutamylphosphate reductase |
| *gene987* | 98 | 100.0 | EFE73306.1 | cysteine synthase |
| *gene988* | 95 | 98.6 | EFE73307.1 | ornithine cyclodeaminase |
| *gene989* | 91 | 101.2 | EFE73308.1 | non-ribosomal peptide synthetase |
| *gene990* | 90 | 100.0 | EFE73309.1 | conserved hypothetical protein |
| *gene991* | 91 | 100.0 | EFE73310.1 | conserved hypothetical protein |
| *gene992* | 96 | 88.0 | EFE73312.1 | nonribosomal peptide synthetase |
| *gene993* | 93 | 94.8 | EFE73312.1 | nonribosomal peptide synthetase |
| *gene994* | 92 | 87.6 | EFE73312.1 | nonribosomal peptide synthetase |
| *gene995* | 91 | 100.0 | EFE73312.1 | nonribosomal peptide synthetase |
| *gene996* | 92 | 96.2 | EFE73312.1 | nonribosomal peptide synthetase |
| *gene997* | 88 | 97.1 | EFE73313.1 | nonribosomal peptide synthetase |
| *gene998* | 92 | 100.4 | EFE73313.1 | nonribosomal peptide synthetase |
| *gene999* | 94 | 97.7 | EFE73314.1 | pyridoxal-5'-phosphate-dependent enzyme |

#### Table S8 Deduced gene functions of *Cluster20*

| Gene | Identity | Coverage | Subject gene | function |
| --- | --- | --- | --- | --- |
| *gene5506* | 53 | 101.6 | BAP34706.1 | LuxR-family transcriptional regulator |
| *gene5507* | 77 | 96.6 | BAP34705.1 | NAD-dependent epimerase/dehydratase |
| *gene5508* | 73 | 94.7 | BAP34704.1 | UDP-glucose/GDP-mannose dehydrogenase |
| *gene5509* | 64 | 96.9 | BAP34703.1 | LmbE family protein |
| *gene5510* | 62 | 103.4 | BAP34763.1 | type I polyketide synthase |
| *gene5515* | 63 | 106.0 | BAP34740.1 | type I polyketide synthase |
| *gene5516* | 62 | 89.1 | BAP34739.1 | type I polyketide synthase |
| *gene5517* | 59 | 105.5 | BAP34738.1 | regulatory protein TetR |
| *gene5518* | 72 | 94.9 | BAP34737.1 | ABC-type multidrug transport system |
| *gene5519* | 68 | 100.0 | BAP34736.1 | ABC-type multidrug transport system |
| *gene5520* | 60 | 97.7 | BAP34735.1 | thioesterase involved in non-ribosomal peptide biosynthesis |
| *gene5521* | 61 | 101.9 | BAP34734.1 | type I polyketide synthase |
| *gene5522* | 64 | 91.6 | BAP34733.1 | type I polyketide synthase |
| *gene5523* | 58 | 97.5 | BAP34733.1 | type I polyketide synthase |
| *gene5524* | 59 | 100.0 | BAP34733.1 | type I polyketide synthase |
| *gene5525* | 81 | 100.8 | BAP34719.1 | cytochrome P450 |
| *gene5526* | 66 | 93.9 | BAP34718.1 | AMP-dependent synthetase and ligase |
| *gene5527* | 58 | 100.0 | BAP34717.1 | acyl carrier protein |
| *gene5528* | 70 | 94.3 | BAP34707.1 | AMP-dependent synthetase and ligase |
| *gene5529* | 68 | 94.7 | BAP34708.1 | malonyl-CoA-ACP transacylase |
| *gene5530* | 73 | 97.3 | BAP34709.1 | (PLP)-dependent aspartate aminotransferase superfamily |
| *gene5531* | 75 | 97.3 | BAP34710.1 | radical-SAM L-lysine 2,3-aminomutase |
| *gene5532* | 75 | 98.7 | BAP34711.1 | proline iminopeptidase |
| *gene5533* | 77 | 98.4 | BAP34712.1 | glycosyltransferase |
| *gene5534* | 64 | 99.7 | BAP34715.1 | glucose-1-phosphate thymidylyltransferase |
| *gene5535* | 66 | 91.7 | BAP34714.1 | dTDP-glucose 4,6-dehydratase |
| *gene5536* | 48 | 98.7 | BAP34742.1 | NDP-hexose 2,3-dehydratase |
| *gene5537* | 59 | 97.5 | BAP34716.1 | N-methyltransferase |
| *gene5538* | 46 | 84.8 | BAP34741.1 | glycosyltransferase |
| *gene5540* | 46 | 99.3 | BAP34747.1 | cytochrome P450 |
| *gene5541* | 61 | 98.8 | BAP34746.1 | glycosyltransferase |
| *gene5542* | 68 | 96.2 | BAP34743.1 | NDP-hexose-3-ketoreductase |
| *gene5543* | 73 | 98.5 | BAP34745.1 | aminotransferase |

#### Table S9 Analysis of transporters according to TransportDB transporter family in the genomes of *Streptomyces* sp. S063 and its related strains

| Family | 063 | 1 | 2 | 3 | 4 | 5 | 6 | 7 | 8 | 9 | 10 | 11 | 12 | 13 | 14 | 15 | 16 | 17 | 18 | 19 | 20 | 21 |
| --- | --- | --- | --- | --- | --- | --- | --- | --- | --- | --- | --- | --- | --- | --- | --- | --- | --- | --- | --- | --- | --- | --- |
| SSS | 1 | 3 | 2 | 4 | 2 | 2 | 2 | 3 | 2 | 7 | 6 | 6 | 2 | 0 | 3 | 0 | 0 | 1 | 0 | 1 | 1 | 1 |
| Nramp | 1 | 3 | 2 | 1 | 1 | 1 | 1 | 1 | 1 | 5 | 4 | 4 | 5 | 2 | 3 | 1 | 2 | 4 | 1 | 1 | 2 | 1 |
| TRAP-T | 0 | 1 | 2 | 1 | 1 | 1 | 1 | 1 | 1 | 4 | 4 | 4 | 4 | 4 | 4 | 1 | 1 | 1 | 1 | 1 | 1 | 1 |
| BCCT | 12 | 4 | 4 | 1 | 2 | 3 | 3 | 3 | 3 | 3 | 1 | 1 | 1 | 1 | 2 | 1 | 2 | 2 | 2 | 2 | 2 | 1 |
| Trk | 3 | 1 | 1 | 1 | 1 | 2 | 2 | 3 | 3 | 1 | 1 | 1 | 1 | 1 | 3 | 1 | 3 | 3 | 1 | 1 | 1 | 1 |
| Tat | 3 | 2 | 2 | 1 | 5 | 5 | 5 | 2 | 2 | 3 | 3 | 3 | 1 | 1 | 1 | 2 | 2 | 2 | 0 | 0 | 0 | 0 |
| MOP | 5 | 3 | 3 | 1 | 2 | 4 | 4 | 1 | 1 | 5 | 3 | 3 | 1 | 0 | 2 | 2 | 0 | 3 | 1 | 0 | 0 | 0 |
| NSS | 0 | 1 | 1 | 0 | 1 | 1 | 1 | 1 | 1 | 1 | 0 | 0 | 0 | 0 | 0 | 1 | 1 | 1 | 0 | 1 | 0 | 0 |
| RhtB | 1 | 0 | 1 | 1 | 3 | 2 | 2 | 1 | 1 | 1 | 1 | 1 | 0 | 0 | 1 | 0 | 1 | 0 | 0 | 0 | 0 | 0 |
| ArsB | 0 | 0 | 0 | 0 | 0 | 1 | 1 | 0 | 0 | 1 | 1 | 1 | 1 | 0 | 0 | 0 | 0 | 0 | 0 | 0 | 0 | 0 |
| ThrE | 1 | 1 | 1 | 0 | 1 | 1 | 1 | 0 | 0 | 1 | 1 | 1 | 0 | 0 | 0 | 0 | 0 | 0 | 0 | 0 | 0 | 0 |
| LIV-E | 0 | 0 | 0 | 0 | 1 | 1 | 1 | 0 | 0 | 1 | 1 | 1 | 0 | 0 | 0 | 0 | 0 | 0 | 0 | 0 | 0 | 0 |
| DAACS | 0 | 0 | 0 | 0 | 1 | 1 | 1 | 0 | 0 | 1 | 1 | 1 | 0 | 0 | 0 | 0 | 0 | 0 | 0 | 0 | 0 | 0 |
| LctP | 0 | 0 | 0 | 0 | 1 | 1 | 1 | 0 | 0 | 2 | 1 | 1 | 0 | 0 | 0 | 0 | 0 | 0 | 0 | 0 | 0 | 0 |
| MscL | 2 | 0 | 0 | 0 | 2 | 1 | 1 | 0 | 0 | 1 | 1 | 1 | 0 | 0 | 0 | 0 | 0 | 0 | 0 | 0 | 0 | 0 |
| GPTS | 2 | 0 | 1 | 0 | 2 | 2 | 1 | 0 | 0 | 2 | 2 | 2 | 0 | 0 | 0 | 0 | 0 | 0 | 0 | 0 | 0 | 0 |
| ACR3 | 1 | 0 | 0 | 0 | 1 | 1 | 1 | 0 | 0 | 1 | 1 | 1 | 0 | 0 | 0 | 0 | 0 | 0 | 0 | 0 | 0 | 0 |
| NhaA | 10 | 0 | 0 | 0 | 3 | 3 | 3 | 0 | 0 | 2 | 0 | 0 | 0 | 0 | 0 | 1 | 0 | 0 | 0 | 0 | 0 | 0 |
| NCS1 | 0 | 0 | 1 | 6 | 4 | 2 | 2 | 2 | 2 | 3 | 1 | 1 | 1 | 0 | 0 | 0 | 0 | 0 | 0 | 0 | 1 | 0 |
| POT | 0 | 0 | 0 | 0 | 2 | 2 | 2 | 0 | 0 | 3 | 1 | 1 | 0 | 0 | 0 | 0 | 0 | 0 | 0 | 0 | 0 | 0 |
| MscS | 1 | 0 | 0 | 0 | 3 | 3 | 2 | 0 | 0 | 4 | 2 | 2 | 0 | 0 | 0 | 0 | 0 | 0 | 1 | 1 | 0 | 0 |
| Amt | 0 | 0 | 0 | 0 | 3 | 3 | 3 | 0 | 0 | 2 | 2 | 2 | 0 | 0 | 0 | 0 | 0 | 0 | 0 | 0 | 0 | 0 |
| TTT | 0 | 0 | 0 | 1 | 4 | 2 | 2 | 0 | 0 | 5 | 2 | 3 | 1 | 1 | 1 | 1 | 1 | 2 | 0 | 0 | 0 | 0 |
| GntP | 2 | 0 | 0 | 0 | 2 | 2 | 2 | 0 | 0 | 5 | 3 | 3 | 0 | 0 | 0 | 0 | 0 | 0 | 0 | 0 | 0 | 0 |

Note: Marine isolated strain was marked in light blue; abundance color of the marine adaption genes: blue, 0; yellow, middle; red, 10 or more.

Strain description: 063, *Streptomyces* sp. S063; 1, *S. sulphureus* DSM 40104; 2, *S. sulphureus* L180; 3, *Streptomyces* sp. CNT360; 4, *S. nanshensis* 10399; 5, *S. qinglanensis* 10379; 6, *S. nanshensis* 10374; 7, *Streptomyces* sp. AA1529; 8, *Streptomyces* sp. CNT318; 9, *S. nanshensis* 01066; 10, *S. abyssalis* 10389; 11, *S. abyssalis* 10390; 12, *Streptomyces* sp. TAA486; 13, *S. nanshensis* 10429; 14, *Streptomyces* sp. TAA204; 15, *Streptomyces* sp. CNH287; 16, *S. oceani* 02100; 17, *Streptomyces* sp. CNS606; 18, *Streptomyces* sp. W007; 19, *Streptomyces* sp. CNB091; 20, *S. griseus* NBRC 13350; 21, *S. fulvissimus* DSM 40593.

MAT family: SSS, sodium symporter; Nramp, metal ion (Mn^2+^) transporter; TRAP-T, tripartite ATP-independent periplasmic transporters; BCCT, betaine/carnitine/choline transporter; Trk, K^+^ transporter; Tat, twin arginine targeting; MOP, multidrug/oligosaccharidyl-lipid/polysaccharides; NSS, neurotransmitter: sodium symporter; RhtB, resistance to homoserine/threonine; ArsB, arsenite-antimonite; ThrE, threonine/serine exporter; LIV-E, branched chain amino acid exporter; DAACS, dicarboxylate/amino acid: cation (Na^+^ or H^+^) symporter; LctP, lactate permease; MscL, large conductance mechanosensitive ion channel; GPTS, general phosphotransferase system; ACR3, arsenical resistance-3; NhaA, Na^+^: H^+^ antiporters; NCS1, the nucleobase: cation symporter-1; POT, proton-dependent oligopeptide transporter; MscS, small conductance mechanosensitive ion channel; Amt, ammonia transporter channel; TTT, tripartite tricarboxylate transporters; GntP, gluconate: H^+^ symporter.

## Supplementary figure legends

#### Figure S1 Comparison of the genome of *Streptomyces* sp. S063 and the reference genomes.

#### Circles from inside to outside: Circle 1, gene location; Circle 2, GC Content; Circle 3, GC Skew; Circle 4-11, genome of *S. griseus* NBRC 13350, *S. fulvissimus* DSM 40593, *Streptomyces* sp. SirexAA-E, *S. venezuelae* ATCC 10712, *S. avermitillis* MA-4680, *S. albus* J1074, *S. scabiei* 87-22, *S. bingchenggensis* BCW-1; Circle 12-15, genome of marine-derived strains, *Streptomyces* sp. CNQ-509, *Streptomyces* sp. SCSIO 03032, *Streptomyces* sp. PVA 94-07, *Streptomyces* sp. PVA 94-10. This comparison figure was built by BLAST Ring Image Generator (BRIG) version 0.95.

#### Figure S2 Neighbor-joining tree showing the phylogenetic relationships of *Streptomyces* sp. S063 with the related reference strains based on 16S-23S rRNA internal transcribed spacer sequences.

The sequences were selected based on the alignment from BLAST and the NJTree was constructed by Geneious. Bootstrap values were set as 1000 replicates. Bar, 0.01 substitutions per nucleotide position.

#### Figure S3 The GNPS map of *Streptomyces* sp. S063.

#### The culture collected from A1 agar was cut into small pieces and extracted by MeOH:Water (1:1), ACN:EtOAc (1:1) and EtOAc, respectively. The samples were analyzed by HPLC-MS/MS and the acquired data was upload and compared with GNPS. The generated data from GNPS was visualized by Cytoscape v3.4.


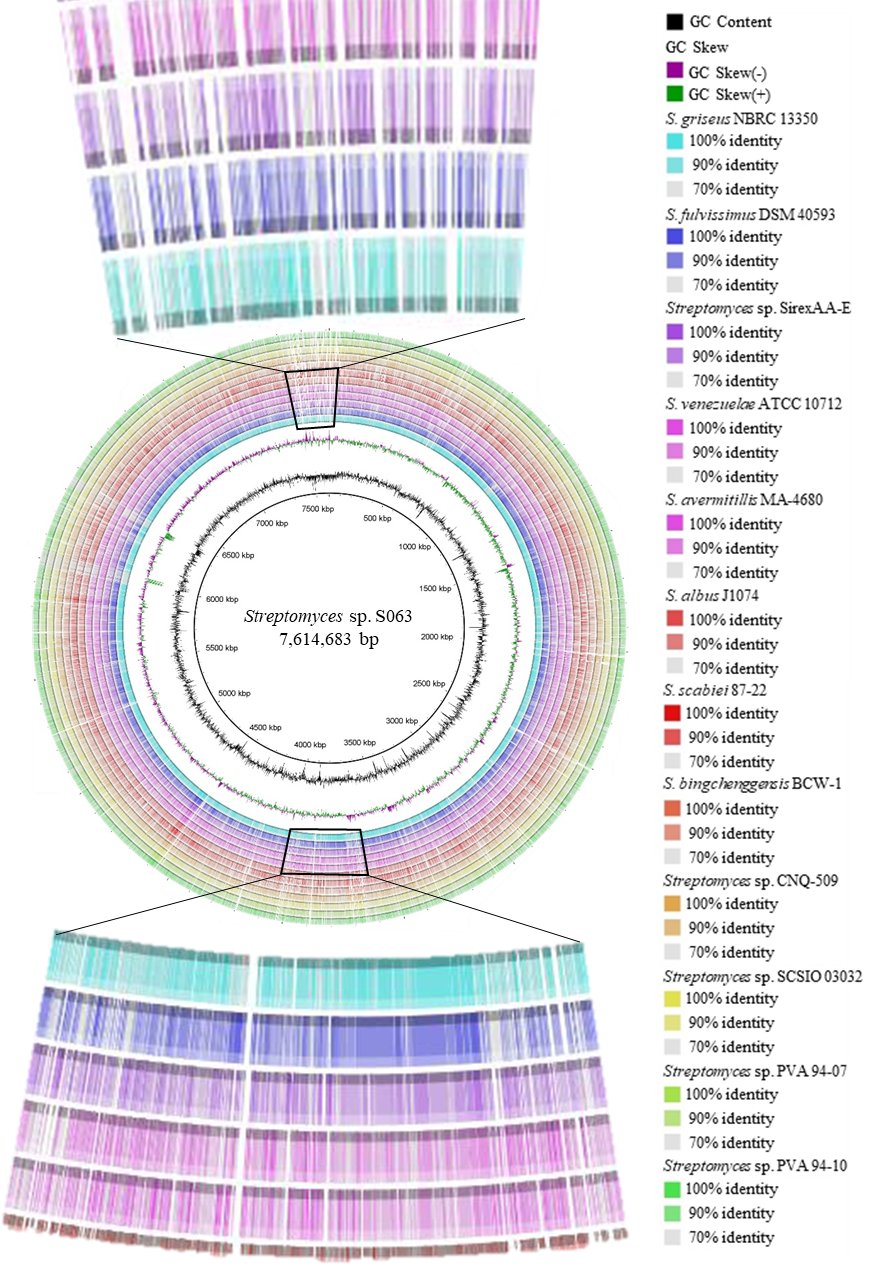


#### Figure S1


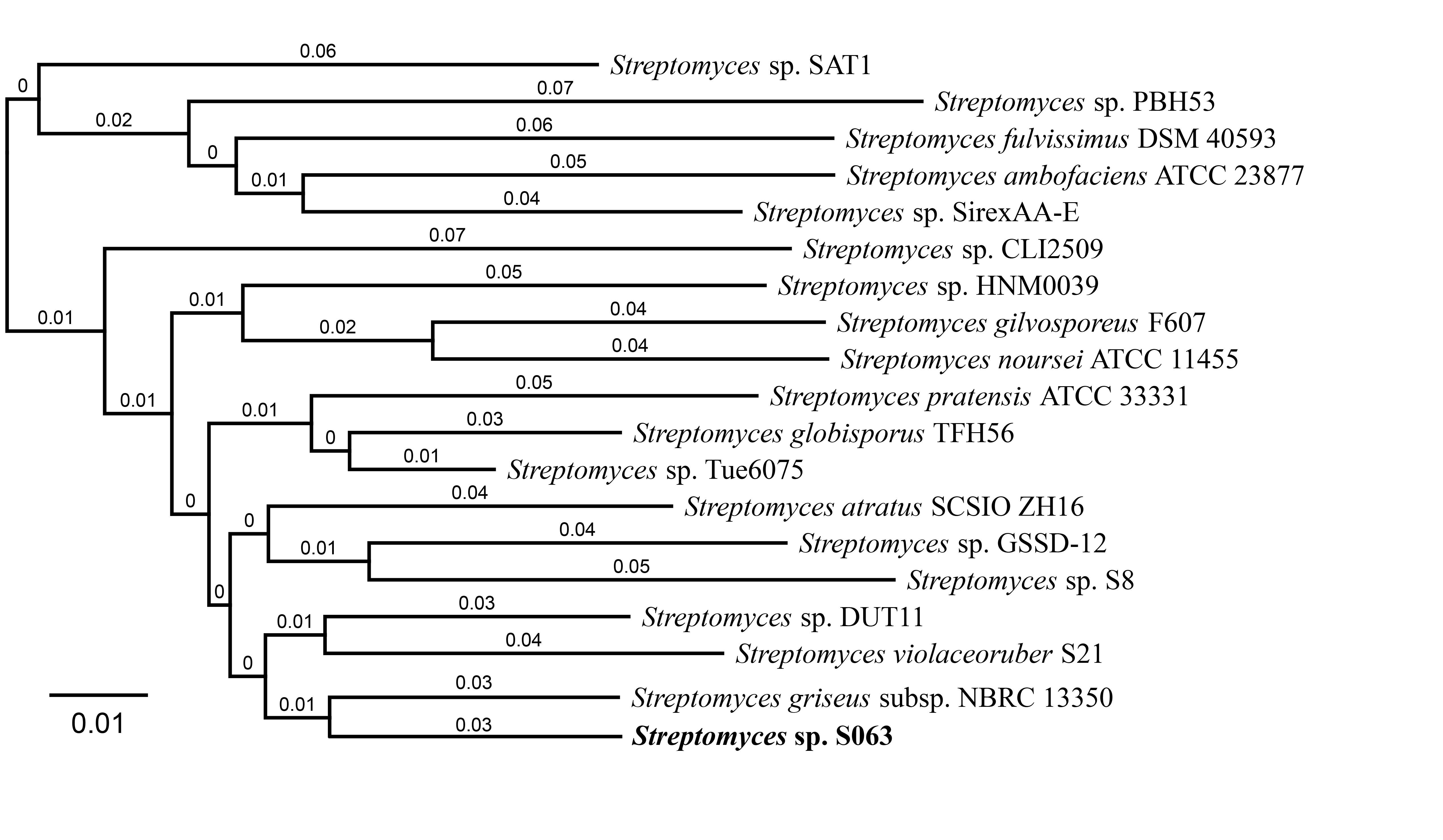


Figure S2


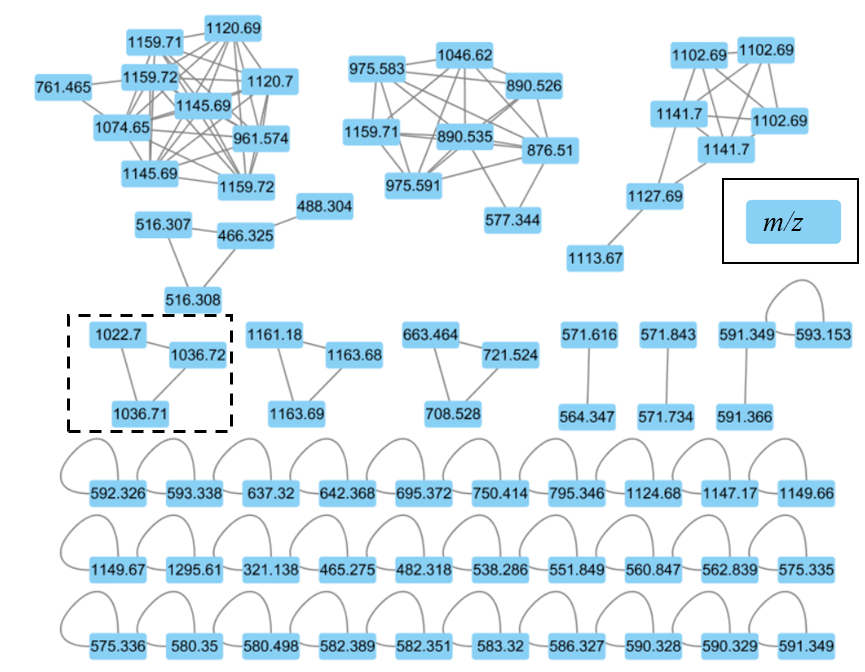


#### Figure S3
